# Metabolic phenotyping of the human microbiome

**DOI:** 10.12688/f1000research.19481.1

**Published:** 2019-11-22

**Authors:** Wiley Barton, Orla O'Sullivan, Paul D. Cotter

**Affiliations:** 1 Department of Food Biosciences, Teagasc Food Research Centre, Moorepark, Fermoy, Cork, P61C996, Ireland; 2 APC Microbiome Ireland, University College Cork, National University of Ireland, Cork, T12YT20, Ireland; 3 VistaMilk SFI Research Centre, Teagasc, Moorepark, Fermoy, Cork, P61C996, Ireland

**Keywords:** microbiome, metagenomics, metabolomics, short chain fatty acids, bile acids

## Abstract

The human microbiome has been identified as having a key role in health and numerous diseases. Trillions of microbial cells and viral particles comprise the microbiome, each representing modifiable working elements of an intricate bioactive ecosystem. The significance of the human microbiome as it relates to human biology has progressed through culture-dependent (for example, media-based methods) and, more recently, molecular (for example, genetic sequencing and metabolomic analysis) techniques. The latter have become increasingly popular and evolved from being used for taxonomic identification of microbiota to elucidation of functional capacity (sequencing) and metabolic activity (metabolomics). This review summarises key elements of the human microbiome and its metabolic capabilities within the context of health and disease.

## Introduction

### The human microbiome as it relates to metabolic function and health

It has been established that communities of microorganisms, *microbiota*, reside on or within nearly every physical substrate on our planet (and associated artificial satellites) ^1– 10^. Composed of organisms encompassing multiple divisions of the tree of life, such as protozoa ^11– 16^, fungi ^17– 20^, viruses ^21– 24^ and prokaryota ^25– 29^, these microbial communities are intricate ecological structures driven by the production and exchange of metabolic products ^29– 34^. Indeed, these communities can cause metabolic cascades that have measurable influences on their macroscopic hosts. Through recognition of these influences, the importance of the microbiome as an integral component of human biology has come to be appreciated, not only by microbiologists but by clinicians and the general public. This review describes essential background to the human microbiome, providing an overview of microbiomes delineated by human anatomy within the framework of microbe–host metabolic interaction before focusing on these interactions as they relate to the gut.

### Womb to tomb

Present from birth to death, an individual’s microbiome maintains a constant presence as a chimeric organ ^35– 38^. Seeding of this microbial system occurs at the beginning of life via transmission of a mother’s microbiome to her infant during the birthing process ^39– 43^. Influenced by direct environmental transmission, a delivered infant will inherit either the mother’s vaginal and faecal microbiota as it passes through the birthing canal or the skin microbiota during caesarean delivery ^39– 41^. Either route of delivery imposes prolonged multifaceted effects on the infant ^44, 45^. Vaginal birth confers a microbiome of the mother’s urogenital system which has undergone specific alterations throughout the pregnancy which are conducive to the development of robust and functional immune and gastrointestinal (GI) systems of the infant ^42^. Alternatively, numerous deleterious health effects for infants delivered by caesarean section have been identified. Immediate influences upon the infant include increased risk of exposure to antibiotic-resistant bacteria from the mother’s skin ^40^. Long-term insults to health arising from caesarean delivery include greater risk of developing obesity, sensitivity to food and inhalant allergens, and asthma ^44– 48^. In light of increasing awareness of potential negative health effects associated with caesarean delivery, an experimental procedure of vaginal seeding has been developed to simulate the microbial exposures present in vaginal birth via administration of vaginal swabs to newly delivered infants ^49^. However, implementing vaginal seeding is a contentious issue, and many clinical practitioners are wary of the intervention prior to extensive investigation of its effects ^50, 51^.

Throughout infancy, an individual’s core microbiome is continuously influenced by the mother and environment. Whether nourished by the mother’s natural breast milk or formula, the infant microbiome continues to be moulded through supplied nutrition. In this regard, a positive health bias towards biological ‘tradition’ persists, as both the process of breast feeding and breast milk itself, and potentially the microbes therein, convey health benefits superior to those of formula ^42, 52, 53^. Progressing through infancy, the microbiome goes through highly variable changes, beginning to stabilise at about 2 years of age. Flux of the microbiome during this period is attributed to numerous factors, including dietary variations (for example, milk versus solid food), immunological development, introduction to novel microbes, and antibiotic exposure ^40, 42, 43, 53– 55^.

Through the transition from infancy to childhood and onto adulthood, the microbiome of an individual stabilises while still being influenced by drug exposure ^29, 56– 59^, physical activity ^60– 70^, the environment ^3^ and diet ^21, 71, 72^ (discussed more elaborately in proceeding sections) ^73, 74^. The microbiome changes again with old age ^75– 77^, and microbes ultimately contribute to decomposition after death ^78– 80^.

## The human body: a microbiome perspective

Microbial communities take form within any accessible area of a host’s body. The defined niches with stable communities in humans and other mammals are currently generalised to the respiratory system ^20, 81– 84^, nasal ^25, 85, 86^ and oral ^17, 25, 26, 87^ cavities, skin ^22, 25, 26, 41, 88– 93^, vagina and urinary tract ^25, 40, 41, 49, 94– 96^, and GI system ^21, 25– 27, 29, 36– 38, 40, 97^. For each of these unique communities, varied challenges are involved in their sampling and analysis and in interpreting their impact on health or disease.

### The skin

**Figure 1.**
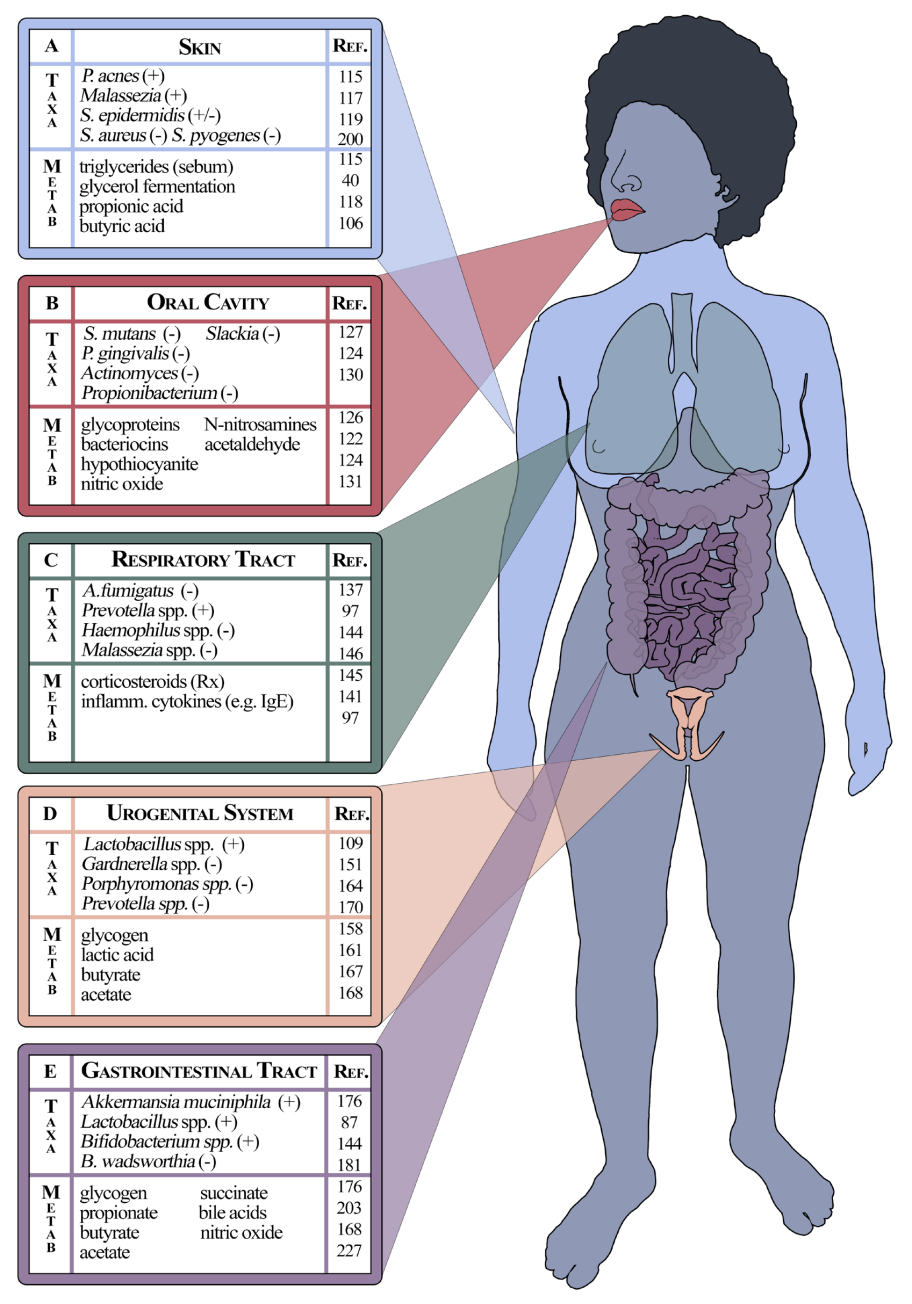
Demonstration of key microbiota and metabolites of the human microbiome, delineated according to human physiology. (A) The skin, (B) oral cavity, (C) respiratory tract, (D) urogenital system and (E) gastrointestinal tract are each highlighted with examples of microbiota (Taxa) and relevant metabolic activity (Metab). Beneficial associations to host health are denoted as (+) and negative associations as (−).

Comprising a relatively large surface area (~1.8 m ^2^ for an adult human) and an array of subsystems defined by folds, crevices, pH, secretion profiles, and environmental exposures, the skin supports highly varied microbial communities functioning in diverse ecological constraints ( Figure 1A) ^89, 98, 99^. Ecological partitioning of the skin microbiome is further defined by elementary biological traits of the host. Microbial composition at specific anatomical locations coordinates with gender ^98, 100, 101^. Indeed, topical sampling of hand palms demonstrates greater diversity of bacterial taxa in women than men, and specific taxa are differentially abundant between the two sexes ^100, 101^. Similar results have been presented for other body sites, such as the thigh and torso ^98, 100^. Expectedly, cohabitation of sexually active partners results in a shared skin microbiome that accurately matches couples 86% of the time ^100^. Ancestral host genetics have also been demonstrated to influence the composition of the skin microbiome. Male participants of diverse ethnic backgrounds, all dwelling in a single geographic location, were shown to have microbial differences specific to ethnicity ^102^. Furthermore, a study of both monozygotic and dizygotic twins described an association between *Corynebacterium jeikeium* and single-nucleotide polymorphisms of a host gene involved in epidermal barrier function ^103^. This finding suggests that the establishment of specific skin microbes is dependent on heritable factors of the host. Despite such associations with the skin microbiome, ancestral genetics have been shown to exert a negligible influence on the gut microbiome, where instead other factors, such as environment, play a more profound role in the form and function of the microbial community ^104^.

Continuous environmental interaction unsurprisingly results in the skin being our most exposed microbial ecosystem. Environmental factors shown to be influential include hygiene routines, topical medication and cosmetic use, and residential environment (for example, rural versus urban) ^89, 91, 98, 101, 105^. Despite its vulnerability to external perturbations, an individual’s skin microbiome maintains a consistent core structure ^106^. Though capable of opportunistic pathogenicity under certain conditions, constituents of this stable community perform homeostatic functions and act as a barrier against transient and potentially pathogenic species, subsequently maintaining a role in a variety of cutaneous conditions ^93, 106– 108^. Among these residential members are strains of *Propionibacterium acnes*, the fungal genus *Malassezia*, and *Staphylococcus epidermidis* ^106, 108– 110^. Lipophilic *P. acnes* and species of *Malassezia* proliferate in sebaceous gland–rich body sites, such as the face and back ^89, 108, 109^. The rich pool of triglycerides found in sebum are hydrolysed by microbes to produce fatty acids that assist in bacterial adherence and maintaining an acidic pH ^108, 111^. Low pH environmental conditions select for lipophilic commensals while inhibiting colonisation by potentially pathogenic strains of *Staphylococcus aureus* and *Staphylococcus pyogenes* ^108, 112^. *P. acnes* additionally contributes to suppression of methicillin-resistant *S. aureus* through glycerol fermentation to short-chain fatty acids (SCFAs) and in particular propionic acid, which also inhibits growth of *Escherichia coli* and *Candida albicans* ^107, 112, 113^.

### The mouth

The oral cavity microbiome represents a reasonably well-defined ecosystem ( Figure 1B). Structure morphology and different tissue types within the human mouth offer a variety of microbial habitats, further delineated by conditions of oxygenation, pH, and nutrient availability ^114, 115^. Control of the oral microbiome is mediated in concert by factors produced by the host and the microbiota ^114, 116– 118^.

Immunological training by microbiota seeded early in life enables the host to distinguish between the commensal core and transient pathogenic microbes, wherein selected commensals create biological barriers through biofilm formation, alter pH and oxygen levels, and produce antimicrobial molecules ^116, 118, 119^. Bacteriocins (that is, small peptide antimicrobials that include the lantibiotics and microcins) are one such means of microbial-derived molecular regulation of community composition within the mouth (and other microbial systems) ^118^. The underlying mechanisms coordinating this antagonistic inter-microbe regulation of community structure require further elucidation; however, its complexity is highlighted by findings of at least 1,169 putative lantibiotic gene clusters within the oral metagenomes defined by the Human Microbiome Project ^120^.

Within this environment, saliva moistens the mouth, aiding in the mastication, swallowing and digestion of food. Saliva also provides an essential nutrient source for microbes, containing complex molecules such as glycoproteins (for example, mucins) ^114, 116, 121, 122^. Similarly, saliva-derived proline-rich glycoproteins contribute to pellicle formation on mouth surfaces, immobilising microbes through their adherence to the structures ^114, 116^. Bioactive compounds found within saliva also include potent factors that inhibit growth or otherwise modify the microbial complex’s activity within the mouth. For example, bacterial growth is curbed by lysozyme-mediated cell lysis and interference of glucose metabolism with lactoperoxidase-catalysed conversion of hydrogen peroxide and thiocyanate to hypothiocyanite ^114, 116^.

Sustaining a balanced oral microbiome is thought to confer numerous local and systemic health benefits. Nitric oxide (NO) is an important cellular signalling molecule, crucially involved with various physiological functions: metabolism, nerve function, and cardiovascular function. Key oral microbiome constituents have demonstrated the ability to reduce dietary nitrates to nitrite ^116, 122, 123^. Converted nitrite is deposited into saliva, which is ingested after oral cavity circulation, leading to NO conversion and the subsequent transmission to tissues across the body ^122, 123^. Countering the potential health benefits of bacterial nitrite supplementation, the compound may stimulate cancer development through formation of carcinogenic N-nitrosamines ^123^. Posing a similar risk of carcinogenesis, acetaldehyde is produced from ethanol by oral bacteria ^122^.

Dysfunction of the oral microbiome contributes directly to dental diseases; the most widely recognised such condition is tooth decay or dental caries. Caries formation begins with bacterial fermentation of carbohydrates to organic acids, resulting in localised pH reduction and subsequent tooth demineralisation ^114, 116, 119, 122^. Once the site has been acidified, the affected environment becomes increasingly selective for bacteria that are tolerant of low pH conditions, thus stimulating proliferation of destructive communities and worsening of the condition ^114, 116, 122^. Although *Streptococcus mutans* is implicated in tooth decay, it is evident that no single organism is the causative agent, and instead polymicrobial activity drives the condition with diverse actors from genera such as *Actinomyces*, *Slackia*, *Propionibacterium* and *Lactobacillus* ^119^.

Periodontal disease is also caused by microorganisms. Prolonged biofilm formation at the interface of gingival tissue and the tooth surface leads to the accumulation of pathogenic bacteria that exacerbate inflammation through cytotoxic compounds such as lipopolysaccharides ^116, 122^. Resultant bleeding from inflammation provides a source of iron from heme, a molecule used by pathogenic microbes (for example, *Porphyromonas gingivalis*) ^116, 122^. Without disruption, periodontitis-associated microbes thrive and, with continued immunological antagonisation of the gingival tissue, contribute to induction of a dysregulated inflammatory response, permanently damaging connective tissue and bone ^116, 122^.

### The nose and respiratory system

At one time, the human lung had been considered a sterile biological system unless challenged with disease. Now, however, it is clear that a respiratory microbiome exists ( Figure 1C).

When healthy, the lung environment reflects many characteristics of the mouth and nose interiors, namely moderate thermal stability, high oxygen availability, mucosa-lined internal surfaces, and a continuous influx of environmental microbes. Despite these similarities, modern investigation of respiratory-related microbes in the lungs projects a microbiome of low phylogenetic diversity ^124– 126^. The simplicity of the lung microbiome contrasts with that of the oral cavity, although the latter acts as a major channel for microbiota translocation, and microaspiration of aerosolised material from the upper respiratory tract and direct migration along the oropharynx mucosa occur ^126, 127^.

Whereas some human microbial communities exhibit high levels of diversity when healthy, presenting associations between disease and reduced diversity, the respiratory microbiome is thought to be more susceptible to malignancy when the complexity of its composition increases ^25, 116, 126, 128, 129^. This is observed as far up in the respiratory system as the nasal cavity, and elevated diversity of the inner nostril is associated with a number of allergies ^100^. Conversely, post-surgical outcome of sinus surgery is better with more diverse sinonasal microbial communities, suggesting an unpredictable complex relationship between upper respiratory tract microbial diversity and health ^130^. Ultimately, caution needs to be used when considering diversity as a marker of health.

A clear association between the lung microbiota and compromised pulmonary health has been demonstrated with asthma, an inflammatory disease ^20, 83, 85, 129, 131, 132^. As is the case for many microbiome–health interactions, evidence supports early-life microbial exposures as being critically influential with respect to respiratory health. Strong epidemiological associations assert an increased risk of inflammatory respiratory disease with caesarean birth and reduced risk from diverse antigen presentation (such as rural and farm exposures) ^46, 47, 133– 135^. More specifically, bacterial species of *Lachnospira*, *Veillonella*, *Faecalibacterium* and *Rothia* were found at low relative abundance in the guts of children deemed to be at higher risk of developing asthma ^135^. Other studies have highlighted differences in community complexity of airways that relate to asthma phenotype ^20, 83, 85, 129, 131, 132^. For example, patients with type 2-high (T2-high) asthma, a form of the disease marked by specific type 2 immunological responses, were shown to have significantly lower diversity of fungal species in airway samples when compared with other patients with asthma ^132^. The same study reported an enrichment of species from the *Trichoderma* fungal genus in T2-high patients. Among the extensive work carried out in characterising the role of microbes in asthma, associations have been made between a deviation from the typical predominance of Bacteroidetes members (for example, species of *Prevotella*) to those of Proteobacteria (for example, *Haemophilus* species) ^83, 136, 137^. Given the observation that Proteobacteria are a predominant component of the skin microbiome, it may be that a detrimental transposition of skin-associated microbiota into the lungs plays some role in the aetiology of the disease ^100, 108^. Although this possibility is intriguing, more robust characterisation of which specific Proteobacteria species are present in the separate sites would be needed to further the theory. Similarly, some analysis of the fungal component of the pulmonary microbiome implicates the presence of *Malassezia* species in asthma ^138^. This fungal species is better known as a factor in atopic and seborrhoeic dermatitis, providing a further potential link between the deleterious translocation of skin microbiota and asthma ^110^. It should be noted that these potential links need to be definitely established.

Although our understanding of the respiratory microbiome’s general role in health is continuing to evolve, there is evidence of compositional alterations in the asthmatic lung microbiome in response to corticosteroid treatment ^131, 137^. Patients with asthma, regardless of whether the asthma is resistant or sensitive to corticosteroid treatment, show reduced Bacteroidetes abundance and increased levels of Proteobacteria and Actinobacteria species ^131^. Additionally, host-derived peripheral blood monocytes from the lungs of corticosteroid-resistant patients had inhibited corticosteroid response when co-cultured with an isolate of *Haemophilus parainfluenzae*, a potential pathogen associated with asthma ** ^131^.

### The vagina and urinary tract

The urogenital microbiome influences female health in a variety of ways. It is also responsible for seeding the microbiome of infants passing through the birth canal in the case of vaginal delivery. The establishment of this microbiome can have lifelong influences on the health of the infant ^43, 44, 139– 141^.

Substantial effort has been put towards characterisation of vaginal microbial components and associated metabolic function ( Figure 1D). The healthy vaginal microbiome is characterised as maintaining low microbial diversity, and *Lactobacillus* species typically dominate ^25, 96, 142^. Disruptions to the healthy vaginal microbiome’s stable low complexity are linked to severity of cervical intra-epithelial neoplasia and bacterial vaginosis (BV), and the latter is also associated with an increased susceptibility to acquiring sexually transmitted infection, pelvic inflammatory disease, and preterm birth ^94, 143– 148^.

*Lactobacillus* dominance of the vaginal microbiome appears to be specific to humans and contrasts greatly with levels found in other animals (>70% and ~1%, respectively) ^149^. Several theories have been proposed for the *Lactobacillus*-centric human vaginal microbiome, including a suggestion of a conserved common function of vaginal microorganisms that in humans happens to be fulfilled by *Lactobacillus* species, and that these species are also adapted to the starch rich diets that are typical of humans ^149^. Indeed, the diet hypothesis further suggests that the high glycogen concentrations found within the human vaginal tract reflect dietary carbohydrate catabolism which is facilitated by abundant salivary amylase levels.

Irrespective of its evolutionary basis, the growth of lactobacilli in the vaginal environment is supported by glycoprotein- and mucin-rich genital fluid and high levels of glycogen and α-amylase, and the latter increases the energy availability of glycogen through its by-products ^149– 151^. With *Lactobacillus* proliferation, the oestrogen-mediated low pH of the vagina is further acidified by microbial-derived lactic acid, which is metabolised from glycogen through anaerobic glycolysis ^152– 157^. Low pH (~3.5) and high lactic acid concentrations contribute in conjunction with cervicovaginal fluid, a highly effective antimicrobial and antiviral medium, to maintain a healthy vaginal environment ^155, 157^. With BV, when the vaginal pH rises (>4.5) and microbial composition shifts away from being *Lactobacillus*-dominant to allow other taxa (such as *Gardnerella*) to proliferate, lactic acid levels drop and a more prominent SCFA profile develops ^155^. Although SCFAs are generally associated with health benefits, particularly in the gut, an undesirable pro-inflammatory response appears to be induced by acetate and butyrate within the vaginal tract ^93, 107, 113, 155, 158, 159^.

The vaginal microbiome appears to considerably influence the efficacy of microbicide HIV prevention therapy ^94^. Tenofovir microbicide gel was 59.2% effective in HIV infection prevention for *Lactobacillus*-dominant vaginal communities, but in individuals with a microbiome containing greater proportions of *Gardnerella*, the prevention rate was only 18% ^94^. Controlled doses of tenofovir administered to patients with either *Gardnerella*- or *Lactobacillus*-oriented microbiomes showed significantly lower concentrations of the drug in *Gardnerella*-dominated vaginal communities; indeed, detected drug concentration negatively correlated with *Gardnerella* abundance ^94^. *In vitro* analysis demonstrated that *Gardnerella* and other BV-associated microbes efficiently metabolised the drug through a cleavage of an oxy-methylphosphonic acid side chain of the compound ^94^.

The male urogenital tract microbiome has received less attention. However, emerging investigation of the subject suggests health-relevant microbial activity within this system. Circumcision significantly modifies microbial composition of the coronal sulci of the penis, decreasing the total microbial load, including anaerobic taxa putatively associated with BV ^160, 161^. Reduced HIV infection rates have independently been associated with circumcision, but the underlying factors of this protective effect are unknown ^162^.

### The gut

Of the microbial communities delineated by human physiology, those associated with the GI system have been investigated with the greatest intensity ( Figure 1E) ^12, 21, 27, 29^.

Microbes travel, generally in a uni-directional manner, through the GI tract within ingested material, and the associated communities follow a gradient of community complexity that peaks in the colon ^163– 165^. Once established, the gut microbiome is subject to influence from a limited number of known factors. Perhaps the factor that most profoundly affects this community is host diet, supplying both microbes and nutrients to influence the microbiome’s function and composition ^55, 72, 159, 166, 167^. Plant-based complex carbohydrates, which intestinal microbiota process with enzymes that are absent from the human host, are one such important dietary factor ^159, 167, 168^. Through metabolism of these polysaccharides, microbial fermentation yields SCFAs, compounds with a broad range of purportedly profound effects on the host ^159, 167, 168^.

In addition to dietary constituents, host-derived metabolites can be used by the gut microbiome ^167, 169– 172^. Examples highlighting this host–microbe interaction include bile acids (BAs), which, once acted upon by bacteria, can trigger complex host–microbe signalling cascades, and intestinal mucins, compounds used by mucin specialists (for example, *Akkermansia muciniphila*), providing protective properties to the host ^167, 169– 173^. It is worth noting that, in addition to drugs explicitly affecting microorganisms (that is, antibiotics), the interaction between other medications and microorganisms can be key, affecting microbe composition and function as well as the pharmacokinetics of the drugs ^171, 174– 177^. Indeed, an *in vitro* screen of more than 1000 pharmaceutical compounds to assess their activity against core representative strains of gut bacteria demonstrated that growth of at least one strain was inhibited by 24% of compounds intended to target human cells ^177^. Similarly, the type 2 diabetes drug metformin was shown to alter both the composition and function of the human intestinal microbiota, resulting in an enrichment of genes associated with SCFA metabolism and faecal concentrations of propionate and butyrate ^176^. However, the specifics of microbial metabolic interactions with metformin have yet to be elucidated.

It should also be noted that drugs of intoxication (for example, alcohol and cannabis) are indicated to interact with the microbiome, although studies in this field are somewhat rare and often limited to non-human animal models ^59, 178– 182^. An exception to the pattern, whereby the gut microbiome of chronic cannabis users was investigated ^181^, revealed that, in comparison with controls, chronic cannabis users had a 13-fold reduction in the ratio of *Prevotella* to *Bacteroides*. Lower *Prevotella* abundance was further associated with poor cognition test performance and reduced mitochondrial ATP production ^181^.

Host behaviour, and more specifically physical exercise and fitness, are also recognised as potential modulators of microbial composition and function ^60– 70^. Illustrating the potential influence of extremes of exercise, professional athletes have been shown to harbour a gut microbiome that exhibits a high compositional diversity of microbial taxa and contains a gene profile with robust potential for environmental energy capture ^60, 63^. More specifically, the gut microbiome of a cohort of professional rugby players, in comparison with age-matched controls with similar body mass index to represent the range of body composition in the athletes, contained greater proportions of metabolic pathways associated with potential health benefits. These pathways ranged from those associated with organic cofactor and antibiotic biosynthesis to degradation and biosynthesis of carbohydrates. Such biosynthetic pathways could result in an increased capacity for energy utilisation by the microbiome ^60^. Metabolomic profiling of the athlete gut microbiome revealed elevated levels of SCFAs, which (as noted above) are metabolites with wide health-associated attributes (detailed further below) and are associated with a lean body composition ^183^. The faecal metabolome of these athletes also exhibited elevated levels of trimethylamine-N-oxide (TMAO), a compound that has been associated with cardiovascular disease and atherosclerosis, although these negative associations have been disputed because of the occurrence of high levels of TMAO in populations with a low occurrence of cardiovascular disease ^184^, and thus the significance of these findings with respect to athletes has yet to be determined. From another study (in this instance, of the microbiome of high-performance cyclists), it was shown that the genus *Prevotella* was significantly associated with reported time of exercising ^68^. The study further revealed higher transcriptional activity of *Methanobrevibacter smithii* genes, particularly those related to methanogenesis, in professional cyclists when compared with amateurs. Investigation of amateur half-marathon runners demonstrated that, through the course of high-intensity running, significant changes occurred in certain taxa (for example, *Coriobacteriaceae*) and metabolites within the gut environment ^70^. Intriguingly, the introduction of exercise as a novel stimulus appears to elicit more subtle changes in the gut microbiome. After undergoing a short period (8 weeks) of moderate-intensity exercise, healthy but inactive adults were shown to exhibit only minor changes in the composition of their gut microbiome ^69^. A separate analysis of a combination of lean and obese individuals undergoing a period of structured exercise conversely asserted that concentrations of faecal SCFAs increased in lean participants following exercise while an obesity-dependent shift in microbial diversity was present after exercise and dissipated after a washout period ^185^. In sum, it is apparent that there remains much to be done to completely understand the mechanisms underlying the interaction of exercise and the gut microbiome.

Gut microbiome analysis is carried out predominantly on the terminal end of the GI tract because of the relative ease with which samples can be non-invasively acquired as stool. These samples provide insight into the intestinal microbiome as excreted samples retain microbial cells and metabolites from the lumen and mucosa, although it is important to note that stool does not provide an exact recapitulation of the intestine’s various subsites ^163, 164, 186^.

## Systemic implication of the gut microbiome in health and disease

**Figure 2.**
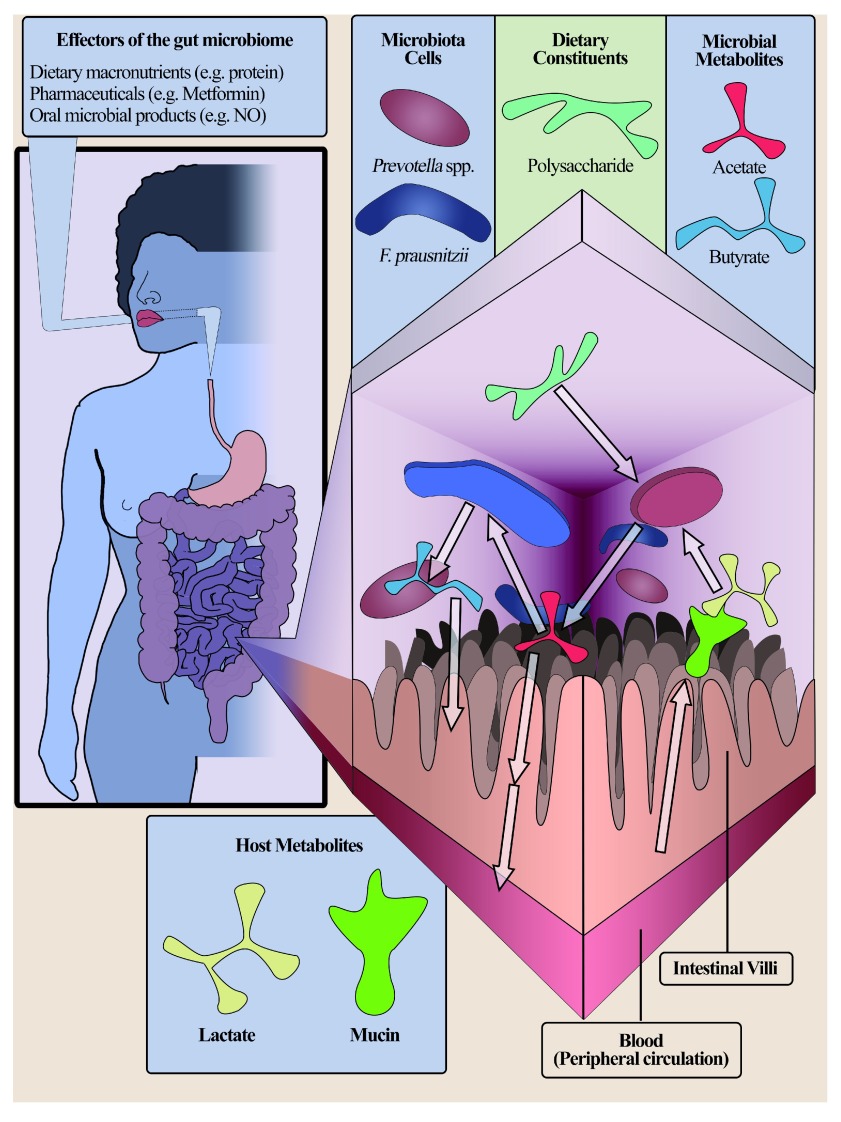
Host–microbe metabolic interaction. A simplified demonstration of the metabolic interactions between host and microbiome. The cross-section of the small intestine illustrates the metabolic exchange between the intestine and two taxonomic representatives ( *Prevotella* spp. and *Faecalibacterium prausnitzii*). Polysaccharides act as an example of dietary substrate used by the microbiota for the production of short-chain fatty acid (butyrate and acetate). Similarly, host-derived substrate in the form of lactate presented with excretion of mucin from the intestine can be used by the microbiota. Within the example, acetate can be either absorbed by the intestine and subsequently the bloodstream where systematic influences take place or converted to butyrate, exerting a localised effect on intestinal epithelial cells. NO, nitric oxide.

The GI system acts as the primary site for the uptake and metabolic processing of nutrients. The gut accordingly contributes substantially to health regulation. As extensive evidence now indicates, intestinal microbes have similar significance in health maintenance and modulation of various disease states via interaction with the host’s biology and intestinal environment. Microbial contributions to this health dynamic are mediated by numerous metabolic modalities. The most prominent such metabolic *circuit* is between the microbiome and ingested nutrients, whereby microbes use dietary nutrients to proliferate and produce metabolites, such as SCFAs, that are involved in cross-talk with the host ( Figure 2) ^29, 37, 72, 166, 167, 187, 188^.

### Short-chain fatty acids

SCFAs act locally within the intestinal system but also impact on hepatic, neurological and immunological function ^158, 159, 188– 192^. As previously noted, microbial SCFA generation results primarily from polysaccharide utilisation, although it has also been demonstrated that some gut microbes have the capacity to produce butyrate from the metabolism of protein ^188, 193– 195^.

Upon excretion from microbial cells, SCFAs entering the intestinal environment are used by colonocytes as an energy source or pass into broader circulation via the portal vein ^159, 188^. Acting locally on colonocytes, butyrate is incorporated into luminal cells through diffusion or direct transport mediated by the Na ^+^-coupled transporter SLC5A8 ^159, 196^. Butyrate within colonocytes contributes to energy production through conversion to acetyl-CoA or alternatively inhibits histone deacetylase (HDAC) activity ^159, 196, 197^. HDAC inhibition occurs within colorectal cancer cells, wherein glucose is preferentially used as an energy source, leading to butyrate accumulation and the subsequent action upon HDAC which results in a cascade of effects on cell proliferation, differentiation and apoptosis ^159, 196, 197^.

Propionate enters systemic circulation through the portal vein, where it is metabolised primarily in the liver while acetate is more broadly circulated, for example, crossing the blood–brain barrier, where it may influence satiety through action on the hypothalamus ^190^. On the basis of murine studies, gut-derived acetate and propionate have separately been suggested to influence asthma ^159, 198, 199^. While regulatory T–cell activity is enhanced by acetate-mediated inhibition of histone deacetylase 9 (HDAC9), resulting in suppression of environmental allergen hypersensitivity, propionate affects lung dendritic cells, dampening promotion of T helper type 2 cell–driven inflammation while leaving the cells’ phagocytic ability intact ^81, 159, 198– 200^.

### Bile acids

BAs have been shown to be at the centre of a metabolic interplay between the host and microbes ^72, 169, 170, 174, 176, 201– 203^. Following post-meal metabolic cues, bile released from the canalicular membrane of hepatocytes enters the intestinal system. Primary BAs, cholic acid and chenodeoxycholic acid are converted from cholesterol and conjugated with taurine or glycine and, within the context of host physiology, are used as detergents to allow intestinal absorption of dietary lipids and fat-soluble vitamins ^201, 203, 204^. Microbial bile salt hydrolases (BSHs) facilitate the hydrolysis of conjugated BAs (CBAs), converting the compounds back to BAs, which permits small intestine reabsorption or additional metabolic processing ^203, 204^. Unconjugated and glycine-CBA absorption by passive diffusion and active transport creates a circulating pool of BAs, establishing continuous bioavailability of the compounds ^202– 204^. As detergents, BAs have the capacity to disrupt the lipid membrane of bacterial cells, subsequently exerting considerable influence on the microbiome. Microbes accordingly employ myriad strategies to circumvent the antimicrobial action of BAs, such as outer membrane lipid and protein modifications ^203, 204^. In conjunction with BA resistance, microbial alterations to BAs, affecting the hydrophobicity of the compounds, also enable some microbes to evade lipid membrane degradation while creating an inhospitable environment for competing organisms ^203, 204^. Microbial BSH-driven hydrolysis of CBAs to unconjugated primary BAs enables subsequent conversion to secondary BAs deoxycholic acid (DCA) and lithocholic acid ^203, 204^. DCA, in particular, accumulates in the enterohepatic BA pool. Relatively high concentrations of DCA result from intestinal diffusion and hepatic reuptake that is facilitated by the compound’s hydrophobicity and the human liver’s inability to rehydroxylate DCA ^203^.

Notably, the fat- and protein-enriched ‘Western’ diet that contributes to obesity development modifies not only gut microbiome composition but also microbial BA pool contributions ^72, 167, 202, 205, 206^. Indeed, the negative consequences of dietary insult have been shown to be ameliorated through intervention with BA-binding resins ^207^. Roux-en-Y gastric bypass surgery has intriguingly been shown to also have an effect on BAs, and serum concentrations are raised in individuals who have undergone the procedure when compared with obese and severely obese controls, suggesting that anatomical manipulation of the procedure modifies the dynamics of the BA pool ^208, 209^.

Among the numerous detrimental effects of obesity, evidence supports a role for microbial-derived DCA as a potent tumour promoter, contributing to the development of hepatocellular carcinoma and the colorectal cancer precursor colorectal adenomas ^72, 202, 210– 212^. Although the associated mechanisms involved have not been studied in the human gut, DCA-driven hepatocellular carcinoma in mice is suggested to result from the compound’s provocation of the senescence-associated secretory phenotype (SASP) in hepatic stellate cells ^211^. SASP is characterised by broad alterations in gene expression and secretory profile, which affect neighbouring cells through numerous factors, namely the release of cytokines (for example, interleukin-1α and -1β), insulin-like growth factor–binding proteins, NO and reactive oxygen species and potentially the glycoprotein fibronectin ^211, 213^. The influence of DCA on colorectal tumorigenesis is proposed to mediate derangement of epidermal growth factor receptor–mitogen-activated protein kinase (EGFR-MAPK) regulation, specifically with DCA preventing degradation of EGFR through calcium signalling of MAPK ^210^. There is still much to be elucidated with respect to the interactions between gut microbes, BAs and health. Furthermore, SCFAs and BAs represent only a small component of the numerous bioactive compounds within the gut environment and thus considerable additional investigation in this area is needed.

## Conclusions and Outlook

Examination of microbiome–host interaction has revealed the integral role of microbiota in health and disease. Extensive characterisation of the microbiome’s taxonomic structure and associations between states of microbial composition and aspects of health have established the groundwork for recognition of the microbiome as a component of human biology. However, the challenge now lies in elucidating the mechanisms underlying the associations between our microbes and health. Metabolic phenotyping and identification of the microbial metabolites interacting with the host will be pivotal to this challenge. With such knowledge, progress can be made in the development of defined microbial cultures (for example, probiotics) and substrates conducive to selective growth or function of microbes (for example, prebiotics) for health enhancement. In short, there is need and opportunity for the innovative deployment of metabolic phenotyping of the human microbiome to develop a new generation of interventions to improve health.

## Abbreviations

BA, bile acid; BSH, bile salt hydrolase; BV, bacterial vaginosis; CBA, conjugated bile acid; DCA, deoxycholic acid; EGFR, epidermal growth factor receptor; GI, gastrointestinal; HDAC, histone deacetylase; MAPK, mitogen-activated protein kinase; NO, nitric oxide; SASP, senescence-associated secretory phenotype; SCFA, short-chain fatty acid; T2-high, type 2-high; TMAO, trimethylamine-N-oxide

## References

[1] Xie W Wang F Guo L: Comparative metagenomics of microbial communities inhabiting deep-sea hydrothermal vent chimneys with contrasting chemistries. *ISME J.* 2011;5(3):414–26. 10.1038/ismej.2010.144 20927138 PMC3105715

[2] Afshinnekoo E Meydan C Chowdhury S: Geospatial Resolution of Human and Bacterial Diversity with City-Scale Metagenomics. *Cell Syst.* 2015;1(1):72–87. 10.1016/j.cels.2015.01.001 26594662 PMC4651444

[3] Ruiz-Calderon JF Cavallin H Song SJ: Walls talk: Microbial biogeography of homes spanning urbanization. *Sci Adv.* 2016;2(2):e1501061. 10.1126/sciadv.1501061 26933683 PMC4758746

[4] Coughlan LM Cotter PD Hill C: New Weapons to Fight Old Enemies: Novel Strategies for the (Bio)control of Bacterial Biofilms in the Food Industry. *Front Microbiol.* 2016;7:1641. 10.3389/fmicb.2016.01641 27803696 PMC5067414

[5] Bourrie BC Willing BP Cotter PD: The Microbiota and Health Promoting Characteristics of the Fermented Beverage Kefir. *Front Microbiol.* 2016;7:647. 10.3389/fmicb.2016.00647 27199969 PMC4854945

[6] Doyle CJ Gleeson D O'Toole PW: High-throughput metataxonomic characterization of the raw milk microbiota identifies changes reflecting lactation stage and storage conditions. *Int J Food Microbiol.* 2017;255:1–6. 10.1016/j.ijfoodmicro.2017.05.019 28554065

[7] Walsh AM Crispie F Daari K: Strain-Level Metagenomic Analysis of the Fermented Dairy Beverage Nunu Highlights Potential Food Safety Risks. *Appl Environ Microbiol.* 2017;83(16):pii: e01144–17. 10.1128/AEM.01144-17 28625983 PMC5541208

[8] McHugh AJ Feehily C Hill C: Detection and Enumeration of Spore-Forming Bacteria in Powdered Dairy Products. *Front Microbiol.* 2017;8:109. 10.3389/fmicb.2017.00109 28197144 PMC5281614

[9] Venkateswaran K Vaishampayan P Cisneros J: International Space Station environmental microbiome - microbial inventories of ISS filter debris. *Appl Microbiol Biotechnol.* 2014;98(14):6453–66. 10.1007/s00253-014-5650-6 24695826

[10] Be NA Avila-Herrera A Allen JE: Whole metagenome profiles of particulates collected from the International Space Station. *Microbiome.* 2017;5(1):81. 10.1186/s40168-017-0292-4 28716113 PMC5514531

[11] Scanlan PD: Blastocystis: past pitfalls and future perspectives. *Trends Parasitol.* 2012;28(8):327–34. 10.1016/j.pt.2012.05.001 22738855

[12] Scanlan PD Knight R Song SJ: Prevalence and genetic diversity of *Blastocystis* in family units living in the United States. *Infect Genet Evol.* 2016;45:95–7. 10.1016/j.meegid.2016.08.018 27545648

[13] Scanlan PD Stensvold CR Rajilić-Stojanović M: The microbial eukaryote *Blastocystis* is a prevalent and diverse member of the healthy human gut microbiota. *FEMS Microbiol Ecol.* 2014;90(1):326–30. 10.1111/1574-6941.12396 25077936

[14] Burgess SL Gilchrist CA Lynn TC: Parasitic Protozoa and Interactions with the Host Intestinal Microbiota. *Infect Immun.* 2017;85(8):pii: e00101–17. 10.1128/IAI.00101-17 28584161 PMC5520446

[15] Chudnovskiy A Mortha A Kana V: Host-Protozoan Interactions Protect from Mucosal Infections through Activation of the Inflammasome. *Cell.* 2016;167(2):444–456.e14. 10.1016/j.cell.2016.08.076 27716507 PMC5129837

[16] Hanevik K Dizdar V Langeland N: Development of functional gastrointestinal disorders after *Giardia lamblia* infection. *BMC Gastroenterol.* 2009;9:27. 10.1186/1471-230X-9-27 19383162 PMC2676300

[17] Ghannoum MA Jurevic RJ Mukherjee PK: Characterization of the oral fungal microbiome (mycobiome) in healthy individuals. *PLoS Pathog.* 2010;6(1):e1000713. 10.1371/journal.ppat.1000713 20072605 PMC2795202

[18] Huffnagle GB Noverr MC: The emerging world of the fungal microbiome. *Trends Microbiol.* 2013;21(7):334–41. 10.1016/j.tim.2013.04.002 23685069 PMC3708484

[19] Huseyin CE O'Toole PW Cotter PD: Forgotten fungi-the gut mycobiome in human health and disease. *FEMS Microbiol Rev.* 2017;41(4):479–511. 10.1093/femsre/fuw047 28430946

[20] Nguyen LD Viscogliosi E Delhaes L: The lung mycobiome: an emerging field of the human respiratory microbiome. *Front Microbiol.* 2015;6:89. 10.3389/fmicb.2015.00089 25762987 PMC4327734

[21] Minot S Sinha R Chen J: The human gut virome: inter-individual variation and dynamic response to diet. *Genome Res.* 2011;21(10):1616–25. 10.1101/gr.122705.111 21880779 PMC3202279

[22] Hannigan GD Zheng Q Meisel JS: Evolutionary and functional implications of hypervariable loci within the skin virome. *PeerJ.* 2017;5:e2959. 10.7717/peerj.2959 28194314 PMC5299996

[23] Virgin HW: The virome in mammalian physiology and disease. *Cell.* 2014;157(1):142–50. 10.1016/j.cell.2014.02.032 24679532 PMC3977141

[24] Norman JM Handley SA Baldridge MT: Disease-specific alterations in the enteric virome in inflammatory bowel disease. *Cell.* 2015;160(3):447–60. 10.1016/j.cell.2015.01.002 25619688 PMC4312520

[25] Human Microbiome Project Consortium: Structure, function and diversity of the healthy human microbiome. *Nature.* 2012;486(7402):207–14. 10.1038/nature11234 22699609 PMC3564958

[26] Costello EK Lauber CL Hamady M: Bacterial community variation in human body habitats across space and time. *Science.* 2009;326(5960):1694–7. 10.1126/science.1177486 19892944 PMC3602444

[27] Gill SR Pop M DeBoy RT: Metagenomic analysis of the human distal gut microbiome. *Science.* 2006;312(5778):1355–9. 10.1126/science.1124234 16741115 PMC3027896

[28] Turnbaugh PJ Hamady M Yatsunenko T: A core gut microbiome in obese and lean twins. *Nature.* 2009;457(7228):480–4. 10.1038/nature07540 19043404 PMC2677729

[29] Zhernakova A Kurilshikov A Bonder MJ: Population-based metagenomics analysis reveals markers for gut microbiome composition and diversity. *Science.* 2016;352(6285):565–9. 10.1126/science.aad3369 27126040 PMC5240844

[30] Scanlan PD: Bacteria-Bacteriophage Coevolution in the Human Gut: Implications for Microbial Diversity and Functionality. *Trends Microbiol.* 2017;25(8):614–23. 10.1016/j.tim.2017.02.012 28342597

[31] Coyte KZ Schluter J Foster KR: The ecology of the microbiome: Networks, competition, and stability. *Science.* 2015;350(6261):663–6. 10.1126/science.aad2602 26542567

[32] Kennedy MJ Volz PA: Ecology of Candida albicans gut colonization: inhibition of Candida adhesion, colonization, and dissemination from the gastrointestinal tract by bacterial antagonism. *Infect Immun.* 1985;49(3):654–63. 3897061 10.1128/iai.49.3.654-663.1985 PMC261235

[33] Smillie CS Smith MB Friedman J: Ecology drives a global network of gene exchange connecting the human microbiome. *Nature.* 2011;480(7376):241–4. 10.1038/nature10571 22037308

[34] Walter J Ley R: The human gut microbiome: ecology and recent evolutionary changes. *Annu Rev Microbiol.* 2011;65:411–29. 10.1146/annurev-micro-090110-102830 21682646

[35] Clarke G Stilling RM Kennedy PJ: Minireview: Gut microbiota: the neglected endocrine organ. *Mol Endocrinol.* 2014;28(8):1221–38. 10.1210/me.2014-1108 24892638 PMC5414803

[36] Evans JM Morris LS Marchesi JR: The gut microbiome: the role of a virtual organ in the endocrinology of the host. *J Endocrinol.* 2013;218(3):R37–R47. 10.1530/JOE-13-0131 23833275

[37] Possemiers S Bolca S Verstraete W: The intestinal microbiome: a separate organ inside the body with the metabolic potential to influence the bioactivity of botanicals. *Fitoterapia.* 2011;82(1):53–66. 10.1016/j.fitote.2010.07.012 20655994

[38] Eckburg PB Bik EM Bernstein CN: Diversity of the human intestinal microbial flora. *Science.* 2005;308(5728):1635–8. 10.1126/science.1110591 15831718 PMC1395357

[39] Dominguez-Bello MG Costello EK Contreras M: Delivery mode shapes the acquisition and structure of the initial microbiota across multiple body habitats in newborns. *Proc Natl Acad Sci U S A.* 2010;107(26):11971–5. 10.1073/pnas.1002601107 20566857 PMC2900693

[40] Bäckhed F Roswall J Peng Y: Dynamics and Stabilization of the Human Gut Microbiome during the First Year of Life. *Cell Host Microbe.* 2015;17(5):690–703. 10.1016/j.chom.2015.04.004 25974306

[41] Chu DM Ma J Prince AL: Maturation of the infant microbiome community structure and function across multiple body sites and in relation to mode of delivery. *Nat Med.* 2017;23(3):314–26. 10.1038/nm.4272 28112736 PMC5345907

[42] Nuriel-Ohayon M Neuman H Koren O: Microbial Changes during Pregnancy, Birth, and Infancy. *Front Microbiol.* 2016;7:1031. 10.3389/fmicb.2016.01031 27471494 PMC4943946

[43] Korpela K Costea P Coelho LP: Selective maternal seeding and environment shape the human gut microbiome. *Genome Res.* 2018;28(4):561–8. 10.1101/gr.233940.117 29496731 PMC5880245

[44] Mueller NT Whyatt R Hoepner L: Prenatal exposure to antibiotics, cesarean section and risk of childhood obesity. *Int J Obes (Lond).* 2015;39(4):665–70. 10.1038/ijo.2014.180 25298276 PMC4390478

[45] Sevelsted A Stokholm J Bønnelykke K: Cesarean section and chronic immune disorders. *Pediatrics.* 2015;135(1):e92–8. 10.1542/peds.2014-0596 25452656

[46] Bager P Wohlfahrt J Westergaard T: Caesarean delivery and risk of atopy and allergic disease: meta-analyses. *Clin Exp Allergy.* 2008;38(4):634–42. 10.1111/j.1365-2222.2008.02939.x 18266879

[47] Negele K Heinrich J Borte M: Mode of delivery and development of atopic disease during the first 2 years of life. *Pediatr Allergy Immunol.* 2004;15(1):48–54. 10.1046/j.0905-6157.2003.00101.x 14998382

[48] Goedert JJ Hua X Yu G: Diversity and composition of the adult fecal microbiome associated with history of cesarean birth or appendectomy: Analysis of the American Gut Project. *eBioMedicine.* 2014;1(2–3):167–72. 10.1016/j.ebiom.2014.11.004 25601913 PMC4296728

[49] Dominguez-Bello MG de Jesus-Laboy KM Shen N: Partial restoration of the microbiota of cesarean-born infants via vaginal microbial transfer. *Nat Med.* 2016;22(3):250–3. 10.1038/nm.4039 26828196 PMC5062956

[50] Haahr T Glavind J Axelsson P: Vaginal seeding or vaginal microbial transfer from the mother to the caesarean-born neonate: a commentary regarding clinical management. *BJOG.* 2018;125(5):533–6. 10.1111/1471-0528.14792 28626982

[51] Cunnington AJ Sim K Deierl A: "Vaginal seeding" of infants born by caesarean section. *BMJ.* 2016;352:i227. 10.1136/bmj.i227 26906151

[52] Cabrera-Rubio R Collado MC Laitinen K: The human milk microbiome changes over lactation and is shaped by maternal weight and mode of delivery. *Am J Clin Nutr.* 2012;96(3):544–51. 10.3945/ajcn.112.037382 22836031

[53] Mueller NT Bakacs E Combellick J: The infant microbiome development: Mom matters. *Trends Mol Med.* 2015;21(2):109–17. 10.1016/j.molmed.2014.12.002 25578246 PMC4464665

[54] Vangay P Ward T Gerber JS: Antibiotics, pediatric dysbiosis, and disease. *Cell Host Microbe.* 2015;17(5):553–64. 10.1016/j.chom.2015.04.006 25974298 PMC5555213

[55] Bokulich NA Chung J Battaglia T: Antibiotics, birth mode, and diet shape microbiome maturation during early life. *Sci Transl Med.* 2016;8(343):343ra82. 10.1126/scitranslmed.aad7121 27306664 PMC5308924

[56] Clooney AG Bernstein CN Leslie WD: A comparison of the gut microbiome between long-term users and non-users of proton pump inhibitors. *Aliment Pharmacol Ther.* 2016;43(9):974–84. 10.1111/apt.13568 26923470

[57] Imhann F Bonder MJ Vich Vila A: Proton pump inhibitors affect the gut microbiome. *Gut.* 2016;65(5):740–8. 10.1136/gutjnl-2015-310376 26657899 PMC4853569

[58] Korpela K Salonen A Virta LJ: Intestinal microbiome is related to lifetime antibiotic use in Finnish pre-school children. *Nat Commun.* 2016;7:10410. 10.1038/ncomms10410 26811868 PMC4737757

[59] Peterson VL Jury NJ Cabrera-Rubio R: Drunk bugs: Chronic vapour alcohol exposure induces marked changes in the gut microbiome in mice. *Behav Brain Res.* 2017;323:172–176. 10.1016/j.bbr.2017.01.049 28161446 PMC5518606

[60] Barton W Penney NC Cronin O: The microbiome of professional athletes differs from that of more sedentary subjects in composition and particularly at the functional metabolic level. *Gut.* 2018;67(4):625–633. 10.1136/gutjnl-2016-313627 28360096

[61] Campbell SC Wisniewski PJ 2nd: Exercise is a Novel Promoter of Intestinal Health and Microbial Diversity. *Exerc Sport Sci Rev.* 2017;45(1):41–7. 10.1249/JES.0000000000000096 27782912

[62] Cerdá B Pérez M Pérez-Santiago JD: Gut Microbiota Modification: Another Piece in the Puzzle of the Benefits of Physical Exercise in Health? *Front Physiol.* 2016;7:51. 10.3389/fphys.2016.00051 26924990 PMC4757670

[63] Clarke SF Murphy EF O'Sullivan O: Exercise and associated dietary extremes impact on gut microbial diversity. *Gut.* 2014;63(12):1913–20. 10.1136/gutjnl-2013-306541 25021423

[64] Cronin O Molloy MG Shanahan F: Exercise, fitness, and the gut. *Curr Opin Gastroenterol.* 2016;32(2):67–73. 10.1097/MOG.0000000000000240 26839963

[65] Mika A Fleshner M: Early-life exercise may promote lasting brain and metabolic health through gut bacterial metabolites. *Immunol Cell Biol.* 2016;94(2):151–7. 10.1038/icb.2015.113 26647967

[66] O'Sullivan O Cronin O Clarke SF: Exercise and the microbiota. *Gut Microbes.* 2015;6(2):131–6. 10.1080/19490976.2015.1011875 25800089 PMC4615660

[67] Paulsen JA Ptacek TS Carter SJ: Gut microbiota composition associated with alterations in cardiorespiratory fitness and psychosocial outcomes among breast cancer survivors. *Support Care Cancer.* 2017;25(5):1563–70. 10.1007/s00520-016-3568-5 28064384 PMC5380600

[68] Petersen LM Bautista EJ Nguyen H: Community characteristics of the gut microbiomes of competitive cyclists. *Microbiome.* 2017;5(1):98. 10.1186/s40168-017-0320-4 28797298 PMC5553673

[69] Cronin O Barton W Skuse P: A Prospective Metagenomic and Metabolomic Analysis of the Impact of Exercise and/or Whey Protein Supplementation on the Gut Microbiome of Sedentary Adults. *mSystems.* 2018;3(3):pii: e00044-18. 10.1128/mSystems.00044-18 29719871 PMC5915698

[70] Zhao X Zhang Z Hu B: Response of Gut Microbiota to Metabolite Changes Induced by Endurance Exercise. *Front Microbiol.* 2018;9:765. 10.3389/fmicb.2018.00765 29731746 PMC5920010

[71] Walsh CJ Guinane CM O'Toole PW: Beneficial modulation of the gut microbiota. *FEBS Lett.* 2014;588(22):4120–30. 10.1016/j.febslet.2014.03.035 24681100

[72] David LA Maurice CF Carmody RN: Diet rapidly and reproducibly alters the human gut microbiome. *Nature.* 2014;505(7484):559–63. 10.1038/nature12820 24336217 PMC3957428

[73] Yatsunenko T Rey FE Manary MJ: Human gut microbiome viewed across age and geography. *Nature.* 2012;486(7402):222–7. 10.1038/nature11053 22699611 PMC3376388

[74] Spor A Koren O Ley R: Unravelling the effects of the environment and host genotype on the gut microbiome. *Nat Rev Microbiol.* 2011;9(4):279–90. 10.1038/nrmicro2540 21407244

[75] Claesson MJ Cusack S O'Sullivan O: Composition, variability, and temporal stability of the intestinal microbiota of the elderly. *Proc Natl Acad Sci U S A.* 2011;108 Suppl 1:4586–91. 10.1073/pnas.1000097107 20571116 PMC3063589

[76] Claesson MJ Jeffery IB Conde S: Gut microbiota composition correlates with diet and health in the elderly. *Nature.* 2012;488(7410):178–84. 10.1038/nature11319 22797518

[77] Salazar N Arboleya S Valdés L: The human intestinal microbiome at extreme ages of life. Dietary intervention as a way to counteract alterations. *Front Genet.* 2014;5:406. 10.3389/fgene.2014.00406 25484891 PMC4240173

[78] Javan GT Finley SJ Abidin Z: The Thanatomicrobiome: A Missing Piece of the Microbial Puzzle of Death. *Front Microbiol.* 2016;7:225. 10.3389/fmicb.2016.00225 26941736 PMC4764706

[79] Javan GT Finley SJ Can I: Human Thanatomicrobiome Succession and Time Since Death. *Sci Rep.* 2016;6:29598. 10.1038/srep29598 27412051 PMC4944132

[80] Metcalf JL Xu ZZ Weiss S: Microbial community assembly and metabolic function during mammalian corpse decomposition. *Science.* 2016;351(6269):158–62. 10.1126/science.aad2646 26657285

[81] Lloyd CM Marsland BJ: Lung Homeostasis: Influence of Age, Microbes, and the Immune System. *Immunity.* 2017;46(4):549–561. 10.1016/j.immuni.2017.04.005 28423336

[82] Madan JC Koestler DC Stanton BA: Serial analysis of the gut and respiratory microbiome in cystic fibrosis in infancy: interaction between intestinal and respiratory tracts and impact of nutritional exposures. *mBio.* 2012;3(4): pii: e00251-1291. 10.1128/mBio.00251-12 22911969 PMC3428694

[83] Millares L Bermudo G Pérez-Brocal V: The respiratory microbiome in bronchial mucosa and secretions from severe IgE-mediated asthma patients. *BMC Microbiol.* 2017;17(1):20. 10.1186/s12866-017-0933-6 28103814 PMC5248442

[84] Morris A Beck JM Schloss PD: Comparison of the respiratory microbiome in healthy nonsmokers and smokers. *Am J Respir Crit Care Med.* 2013;187(10):1067–75. 10.1164/rccm.201210-1913OC 23491408 PMC3734620

[85] Wilson MT Hamilos DL: The nasal and sinus microbiome in health and disease. *Curr Allergy Asthma Rep.* 2014;14(12):485. 10.1007/s11882-014-0485-x 25342392

[86] Liu CM Price LB Hungate BA: *Staphylococcus aureus* and the ecology of the nasal microbiome. *Sci Adv.* 2015;1(5):e1400216. 10.1126/sciadv.1400216 26601194 PMC4640600

[87] Wu J Peters BA Dominianni C: Cigarette smoking and the oral microbiome in a large study of American adults. *ISME J.* 2016;10(10):2435–46. 10.1038/ismej.2016.37 27015003 PMC5030690

[88] Bjerre RD Bandier J Skov L: The role of the skin microbiome in atopic dermatitis: A systematic review. *Br J Dermatol.* 2017;177(5):1272–8. 10.1111/bjd.15390 28207943

[89] Grice EA Kong HH Conlan S: Topographical and Temporal Diversity of the Human Skin Microbiome. *Science.* 2009;324(5931):1190–2. 10.1126/science.1171700 19478181 PMC2805064

[90] Kong HH Andersson B Clavel T: Performing Skin Microbiome Research: A Method to the Madness. *J Invest Dermatol.* 2017;137(37):561–8. 10.1016/j.jid.2016.10.033 28063650 PMC5468751

[91] Kong HH Oh J Deming C: Temporal shifts in the skin microbiome associated with disease flares and treatment in children with atopic dermatitis. *Genome Res.* 2012;22(5):850–9. 10.1101/gr.131029.111 22310478 PMC3337431

[92] Meadow JF Bateman AC Herkert KM: Significant changes in the skin microbiome mediated by the sport of roller derby. *PeerJ.* 2013;1:e53. 10.7717/peerj.53 23638391 PMC3628844

[93] Wang Y Zhang L Yu J: A Co-Drug of Butyric Acid Derived from Fermentation Metabolites of the Human Skin Microbiome Stimulates Adipogenic Differentiation of Adipose-Derived Stem Cells: Implications in Tissue Augmentation. *J Invest Dermatol.* 2017;137(1):46–56. 10.1016/j.jid.2016.07.030 27498050

[94] Klatt NR Cheu R Birse K: Vaginal bacteria modify HIV tenofovir microbicide efficacy in African women. *Science.* 2017;356(6341):938–945. 10.1126/science.aai9383 28572388

[95] Lamont RF Sobel JD Akins RA: The vaginal microbiome: new information about genital tract flora using molecular based techniques. *BJOG.* 2011;118(5):533–49. 10.1111/j.1471-0528.2010.02840.x 21251190 PMC3055920

[96] Ravel J Gajer P Abdo Z: Vaginal microbiome of reproductive-age women. *Proc Natl Acad Sci U S A.* 2011;108 Suppl 1:4680–7. 10.1073/pnas.1002611107 20534435 PMC3063603

[97] Arumugam M Raes J Pelletier E: Enterotypes of the human gut microbiome. *Nature.* 2011;473(7346):174–180. 10.1038/nature09944 21508958 PMC3728647

[98] Ying S Zeng DN Chi L: The Influence of Age and Gender on Skin-Associated Microbial Communities in Urban and Rural Human Populations. *PLoS One.* 2015;10(10):e0141842. 10.1371/journal.pone.0141842 26510185 PMC4624872

[99] Kong HH Segre JA: Skin microbiome: looking back to move forward. *J Invest Dermatol.* 2012;132(3 Pt 2):933–9. 10.1038/jid.2011.417 22189793 PMC3279608

[100] Ross AA Doxey AC Neufeld JD: The Skin Microbiome of Cohabiting Couples. *mSystems.* 2017;2(4):pii: e00043-17. 10.1128/mSystems.00043-17 28761935 PMC5527301

[101] Fierer N Hamady M Lauber CL: The influence of sex, handedness, and washing on the diversity of hand surface bacteria. *Proc Natl Acad Sci U S A.* 2008;105(46):17994–9. 10.1073/pnas.0807920105 19004758 PMC2584711

[102] Perez Perez GI Gao Z Jourdain R: Body Site Is a More Determinant Factor than Human Population Diversity in the Healthy Skin Microbiome. *PLoS One.* 2016;11(4):e0151990. 10.1371/journal.pone.0151990 27088867 PMC4835103

[103] Si J Lee S Park JM: Genetic associations and shared environmental effects on the skin microbiome of Korean twins. *BMC Genomics.* 2015;16:992. 10.1186/s12864-015-2131-y 26596276 PMC4657342

[104] Rothschild D Weissbrod O Barkan E: Environment dominates over host genetics in shaping human gut microbiota. *Nature.* 2018;555(7695):210–5. 10.1038/nature25973 29489753

[105] Urban J Fergus DJ Savage AM: The effect of habitual and experimental antiperspirant and deodorant product use on the armpit microbiome. *PeerJ.* 2016;4:e1605. 10.7717/peerj.1605 26855863 PMC4741080

[106] Oh J Byrd AL Park M: Temporal Stability of the Human Skin Microbiome. *Cell.* 2016;165(4):854–66. 10.1016/j.cell.2016.04.008 27153496 PMC4860256

[107] Wang Y Dai A Huang S: Propionic acid and its esterified derivative suppress the growth of methicillin-resistant Staphylococcus aureus USA300. *Benef Microbes.* 2014;5(2):161–8. 10.3920/BM2013.0031 24686580

[108] Grice EA Segre JA: The skin microbiome. *Nat Rev Micro.* 2011;9(4):244–53. 10.1038/nrmicro2537 21407241 PMC3535073

[109] SanMiguel A Grice EA: Interactions between host factors and the skin microbiome. *Cell Mol Life Sci.* 2015;72(8):1499–515. 10.1007/s00018-014-1812-z 25548803 PMC4376244

[110] Findley K Oh J Yang J: Topographic diversity of fungal and bacterial communities in human skin. *Nature.* 2013;498(7454):367–70. 10.1038/nature12171 23698366 PMC3711185

[111] Gribbon EM Cunliffe WJ Holland KT: Interaction of *Propionibacterium acnes* with skin lipids *in vitro*. *J Gen Microbiol.* 1993;139(8):1745–51. 10.1099/00221287-139-8-1745 8409917

[112] Francuzik W Franke K Schumann RR: Propionibacterium acnes Abundance Correlates Inversely with Staphylococcus aureus: Data from Atopic Dermatitis Skin Microbiome. *Acta Derm Venereol.* 2018;98(5):490–495. 10.2340/00015555-2896 29379979

[113] Noverr MC Huffnagle GB: Regulation of Candida albicans morphogenesis by fatty acid metabolites. *Infect Immun.* 2004;72(11):6206–10. 10.1128/IAI.72.11.6206-6210.2004 15501745 PMC523025

[114] Struzycka I: The oral microbiome in dental caries. *Pol J Microbiol.* 2014;63(2):127–35. 25115106

[115] Krishnan K Chen T Paster BJ: A practical guide to the oral microbiome and its relation to health and disease. *Oral Dis.* 2017;23(3):276–286. 10.1111/odi.12509 27219464 PMC5122475

[116] Kilian M Chapple IL Hannig M: The oral microbiome - an update for oral healthcare professionals. *Br Dent J.* 2016;221(10):657–666. 10.1038/sj.bdj.2016.865 27857087

[117] Liu B Faller LL Klitgord N: Deep sequencing of the oral microbiome reveals signatures of periodontal disease. *PLoS One.* 2012;7(6):e37919. 10.1371/journal.pone.0037919 22675498 PMC3366996

[118] Edlund A Garg N Mohimani H: Metabolic Fingerprints from the Human Oral Microbiome Reveal a Vast Knowledge Gap of Secreted Small Peptidic Molecules. *mSystems.* 2017;2(4): pii: e00058-17. 10.1128/mSystems.00058-17 28761934 PMC5516222

[119] Gomez A Nelson KE: The Oral Microbiome of Children: Development, Disease, and Implications Beyond Oral Health. *Microb Ecol.* 2017;73(2):492–503. 10.1007/s00248-016-0854-1 27628595 PMC5274568

[120] Walsh CJ Guinane CM O’ Toole PW: A Profile Hidden Markov Model to investigate the distribution and frequency of LanB-encoding lantibiotic modification genes in the human oral and gut microbiome. *PeerJ.* 2017;5:e3254. 10.7717/peerj.3254 28462050 PMC5410138

[121] van 't Hof W Veerman EC Nieuw Amerongen AV: Antimicrobial defense systems in saliva. *Monogr Oral Sci.* 2014;24:40–51. 10.1159/000358783 24862593

[122] Takahashi N: Oral Microbiome Metabolism: From "Who Are They?" to "What Are They Doing?". *J Dent Res.* 2015;94(12):1628–37. 10.1177/0022034515606045 26377570

[123] Hezel MP Weitzberg E: The oral microbiome and nitric oxide homoeostasis. *Oral Dis.* 2015;21(1):7–16. 10.1111/odi.12157 23837897

[124] Charlson ES Bittinger K Haas AR: Topographical continuity of bacterial populations in the healthy human respiratory tract. *Am J Respir Crit Care Med.* 2011;184(8):957–63. 10.1164/rccm.201104-0655OC 21680950 PMC3208663

[125] Yu G Gail MH Consonni D: Characterizing human lung tissue microbiota and its relationship to epidemiological and clinical features. *Genome Biol.* 2016;17(1):163. 10.1186/s13059-016-1021-1 27468850 PMC4964003

[126] Dickson RP Huffnagle GB: The Lung Microbiome: New Principles for Respiratory Bacteriology in Health and Disease. *PLoS Pathog.* 2015;11(7):e1004923. 10.1371/journal.ppat.1004923 26158874 PMC4497592

[127] Dickson RP Erb-Downward JR Freeman CM: Bacterial Topography of the Healthy Human Lower Respiratory Tract. *MBio.* 2017;8(1). 10.1128/mBio.02287-16 28196961 PMC5312084

[128] Lozupone CA Stombaugh JI Gordon JI: Diversity, stability and resilience of the human gut microbiota. *Nature.* 2012;489(7415):220–30. 10.1038/nature11550 22972295 PMC3577372

[129] Legatzki A Rösler B von Mutius E: Microbiome diversity and asthma and allergy risk. *Curr Allergy Asthma Rep.* 2014;14(10):466. 10.1007/s11882-014-0466-0 25149168

[130] Ramakrishnan VR Hauser LJ Feazel LM: Sinus microbiota varies among chronic rhinosinusitis phenotypes and predicts surgical outcome. *J Allergy Clin Immunol.* 2015;136(2):334–342.e1. 10.1016/j.jaci.2015.02.008 25819063

[131] Goleva E Jackson LP Harris JK: The effects of airway microbiome on corticosteroid responsiveness in asthma. *Am J Respir Crit Care Med.* 2013;188(10):1193–201. 10.1164/rccm.201304-0775OC 24024497 PMC3863730

[132] Sharma A Laxman B Naureckas ET: Associations between fungal and bacterial microbiota of airways and asthma endotypes. *J Allergy Clin Immunol.* 2019;144(5):1214–1227.e7. 10.1016/j.jaci.2019.06.025 31279011 PMC6842419

[133] Ege MJ Mayer M Normand AC: Exposure to environmental microorganisms and childhood asthma. *N Engl J Med.* 2011;364(8):701–9. 10.1056/NEJMoa1007302 21345099

[134] Stein MM Hrusch CL Gozdz J: Innate Immunity and Asthma Risk in Amish and Hutterite Farm Children. *N Engl J Med.* 2016;375(5):411–421. 10.1056/NEJMoa1508749 27518660 PMC5137793

[135] Arrieta MC Stiemsma LT Dimitriu PA: Early infancy microbial and metabolic alterations affect risk of childhood asthma. *Sci Transl Med.* 2015;7(307):307ra152. 10.1126/scitranslmed.aab2271 26424567

[136] Hilty M Burke C Pedro H: Disordered microbial communities in asthmatic airways. *PLoS One.* 2010;5(1):e8578. 10.1371/journal.pone.0008578 20052417 PMC2798952

[137] Durack J Lynch SV Nariya S: Features of the bronchial bacterial microbiome associated with atopy, asthma, and responsiveness to inhaled corticosteroid treatment. *J Allergy Clin Immunol.* 2017;140(1):63–75. 10.1016/j.jaci.2016.08.055 27838347 PMC5502827

[138] van Woerden HC Gregory C Brown R: Differences in fungi present in induced sputum samples from asthma patients and non-atopic controls: a community based case control study. *BMC Infect Dis.* 2013;13:69. 10.1186/1471-2334-13-69 23384395 PMC3570489

[139] Salam MT Margolis HG McConnell R: Mode of delivery is associated with asthma and allergy occurrences in children. *Ann Epidemiol.* 2006;16(5):341–6. 10.1016/j.annepidem.2005.06.054 16242961

[140] Kero J Gissler M Grönlund M-M: Mode of delivery and asthma -- is there a connection? *Pediatr Res.* 2002;52(1):6–11. 10.1203/00006450-200207000-00004 12084840

[141] Yip BHK Leonard H Stock S: Caesarean section and risk of autism across gestational age: A multi-national cohort study of 5 million births. *Int J Epidemiol.* 2017;46(2):429–39. 10.1093/ije/dyw336 28017932 PMC5837358

[142] Whiteside SA Razvi H Dave S: The microbiome of the urinary tract—a role beyond infection. *Nat Rev Urol.* 2015;12(2):81–90. 10.1038/nrurol.2014.361 25600098

[143] Hillier SL Nugent RP Eschenbach DA: Association between bacterial vaginosis and preterm delivery of a low-birth-weight infant. The Vaginal Infections and Prematurity Study Group. *N Engl J Med.* 1995;333(26):1737–42. 10.1056/NEJM199512283332604 7491137

[144] Mitra A MacIntyre DA Lee YS: Cervical intraepithelial neoplasia disease progression is associated with increased vaginal microbiome diversity. *Sci Rep.* 2015;5:16865 10.1038/srep16865 26574055 PMC4648063

[145] Ness RB Kip KE Hillier SL: A cluster analysis of bacterial vaginosis-associated microflora and pelvic inflammatory disease. *Am J Epidemiol.* 2005;162(6):585–90. 10.1093/aje/kwi243 16093289

[146] Sewankambo N Gray RH Wawer MJ: HIV-1 infection associated with abnormal vaginal flora morphology and bacterial vaginosis. *Lancet.* 1997;350(9077):546–50. 10.1016/s0140-6736(97)01063-5 9284776

[147] Gravett MG: Independent associations of bacterial vaginosis and Chlamydia trachomatis infection with adverse pregnancy outcome. *JAMA.* 1986;256(14):1899–903. 3761496

[148] Ma B Forney LJ Ravel J: Vaginal microbiome: rethinking health and disease. *Annu Rev Microbiol.* 2012;66:371–89. 10.1146/annurev-micro-092611-150157 22746335 PMC3780402

[149] Miller EA Beasley DE Dunn RR: Lactobacilli Dominance and Vaginal pH: Why Is the Human Vaginal Microbiome Unique? *Front Microbiol.* 2016;7:1936. 10.3389/fmicb.2016.01936 28008325 PMC5143676

[150] Boris S Suárez JE Vázquez F: Adherence of human vaginal lactobacilli to vaginal epithelial cells and interaction with uropathogens. *Infect Immun.* 1998;66:1985–9. 9573080 10.1128/iai.66.5.1985-1989.1998 PMC108154

[151] Moncla BJ Chappell CA Debo BM: The Effects of Hormones and Vaginal Microflora on the Glycome of the Female Genital Tract: Cervical-Vaginal Fluid. *PLoS One.* 2016;11(7):e0158687. 10.1371/journal.pone.0158687 27437931 PMC4954690

[152] Gorodeski GI Hopfer U Liu CC: Estrogen acidifies vaginal pH by up-regulation of proton secretion via the apical membrane of vaginal-ectocervical epithelial cells. *Endocrinology.* 2005;146(2):816–24. 10.1210/en.2004-1153 15498880 PMC2398721

[153] Boskey ER Telsch KM Whaley KJ: Acid production by vaginal flora *in vitro* is consistent with the rate and extent of vaginal acidification. *Infect Immun.* 1999;67(10):5170–5. 10496892 10.1128/iai.67.10.5170-5175.1999 PMC96867

[154] Gorodeski GI: Effects of estrogen on proton secretion via the apical membrane in vaginal-ectocervical epithelial cells of postmenopausal women. *Menopause.* 2005;12(6):679–84. 10.1097/01.gme.0000184423.88814.e6 16278610 PMC2373250

[155] Aldunate M Srbinovski D Hearps AC: Antimicrobial and immune modulatory effects of lactic acid and short chain fatty acids produced by vaginal microbiota associated with eubiosis and bacterial vaginosis. *Front Physiol.* 2015;6:164. 10.3389/fphys.2015.00164 26082720 PMC4451362

[156] Linhares IM Summers PR Larsen B: Contemporary perspectives on vaginal pH and lactobacilli. *Am J Obstet Gynecol.* 2011;204(2):120.e1-5. 10.1016/j.ajog.2010.07.010 20832044

[157] O’Hanlon DE Moench TR Cone RA: Vaginal pH and microbicidal lactic acid when lactobacilli dominate the microbiota. *PLoS One.* 2013;8(11):e80074. 10.1371/journal.pone.0080074 24223212 PMC3819307

[158] Mirmonsef P Zariffard MR Gilbert D: Short-chain fatty acids induce pro-inflammatory cytokine production alone and in combination with toll-like receptor ligands. *Am J Reprod Immunol.* 2012;67(5):391–400. 10.1111/j.1600-0897.2011.01089.x 22059850 PMC3288536

[159] Koh A De Vadder F Kovatcheva-Datchary P: From Dietary Fiber to Host Physiology: Short-Chain Fatty Acids as Key Bacterial Metabolites. *Cell.* 2016;165(6):1332–45. 10.1016/j.cell.2016.05.041 27259147

[160] Price LB Liu CM Johnson KE: The effects of circumcision on the penis microbiome. *PLoS One.* 2010;5(1):e8422. 10.1371/journal.pone.0008422 20066050 PMC2798966

[161] Liu CM Hungate BA Tobian AA: Male circumcision significantly reduces prevalence and load of genital anaerobic bacteria. *mBio.* 2013;4(2): e00076. 10.1128/mBio.00076-13 23592260 PMC3634604

[162] Prodger JL Kaul R: The biology of how circumcision reduces HIV susceptibility: broader implications for the prevention field. *AIDS Res Ther.* 2017;14(1):49. 10.1186/s12981-017-0167-6 28893286 PMC5594533

[163] Yasuda K Oh K Ren B: Biogeography of the intestinal mucosal and lumenal microbiome in the rhesus macaque. *Cell Host Microbe.* 2015;17(3):385–91. 10.1016/j.chom.2015.01.015 25732063 PMC4369771

[164] Tropini C Earle KA Huang KC: The Gut Microbiome: Connecting Spatial Organization to Function. *Cell Host Microbe.* 2017;21(4):433–42. 10.1016/j.chom.2017.03.010 28407481 PMC5576359

[165] Bik EM Eckburg PB Gill SR: Molecular analysis of the bacterial microbiota in the human stomach. *Proc Natl Acad Sci U S A.* 2006;103(3):732–7. 10.1073/pnas.0506655103 16407106 PMC1334644

[166] Lang JM Eisen JA Zivkovic AM: The microbes we eat: abundance and taxonomy of microbes consumed in a day's worth of meals for three diet types. *PeerJ.* 2014;2:e659. 10.7717/peerj.659 25538865 PMC4266855

[167] Sonnenburg JL Bäckhed F: Diet-microbiota interactions as moderators of human metabolism. *Nature.* 2016;535(7610):56–64. 10.1038/nature18846 27383980 PMC5991619

[168] Martens EC Kelly AG Tauzin AS: The devil lies in the details: how variations in polysaccharide fine-structure impact the physiology and evolution of gut microbes. *J Mol Biol.* 2014;426(23):3851–65. 10.1016/j.jmb.2014.06.022 25026064 PMC4252772

[169] Jones BV Begley M Hill C: Functional and comparative metagenomic analysis of bile salt hydrolase activity in the human gut microbiome. *Proc Natl Acad Sci U S A.* 2008;105(36):13580–5. 10.1073/pnas.0804437105 18757757 PMC2533232

[170] Joyce SA MacSharry J Casey PG: Regulation of host weight gain and lipid metabolism by bacterial bile acid modification in the gut. *Proc Natl Acad Sci U S A.* 2014;111(20):7421–6. 10.1073/pnas.1323599111 24799697 PMC4034235

[171] Nicholson JK Holmes E Kinross J: Host-gut microbiota metabolic interactions. *Science.* 2012;336(6086):1262–7. 10.1126/science.1223813 22674330

[172] van Passel MW Kant R Zoetendal EG: The genome of *Akkermansia muciniphila*, a dedicated intestinal mucin degrader, and its use in exploring intestinal metagenomes. *PLoS One.* 2011;6(3):e16876. 10.1371/journal.pone.0016876 21390229 PMC3048395

[173] Belzer C Chia LW Aalvink S: Microbial Metabolic Networks at the Mucus Layer Lead to Diet-Independent Butyrate and Vitamin B _12_ Production by Intestinal Symbionts. *mBio.* 2017;8(5): pii: e00770-17. 10.1128/mBio.00770-17 28928206 PMC5605934

[174] Vrieze A Out C Fuentes S: Impact of oral vancomycin on gut microbiota, bile acid metabolism, and insulin sensitivity. *J Hepatol.* 2014;60(4):824–31. 10.1016/j.jhep.2013.11.034 24316517

[175] Enright EF Gahan CG Joyce SA: The Impact of the Gut Microbiota on Drug Metabolism and Clinical Outcome. *Yale J Biol Med.* 2016;89(3):375–82. 27698621 PMC5045146

[176] Wu H Esteve E Tremaroli V: Metformin alters the gut microbiome of individuals with treatment-naive type 2 diabetes, contributing to the therapeutic effects of the drug. *Nat Med.* 2017;23(7):850–8. 10.1038/nm.4345 28530702

[177] Maier L Pruteanu M Kuhn M: Extensive impact of non-antibiotic drugs on human gut bacteria. *Nature.* 2018;555(7698):623–8. 10.1038/nature25979 29555994 PMC6108420

[178] Cluny NL Keenan CM Reimer RA: Prevention of Diet-Induced Obesity Effects on Body Weight and Gut Microbiota in Mice Treated Chronically with Δ ^9^-Tetrahydrocannabinol. *PLoS One.* 2015;10(12):e0144270. 10.1371/journal.pone.0144270 26633823 PMC4669115

[179] Mutlu EA Gillevet PM Rangwala H: Colonic microbiome is altered in alcoholism. *Am J Physiol Gastrointest Liver Physiol.* 2012;302(9):G966–78. 10.1152/ajpgi.00380.2011 22241860 PMC3362077

[180] Wilson ID Nicholson JK: Gut microbiome interactions with drug metabolism, efficacy, and toxicity. *Transl Res.* 2017;179:204–22. 10.1016/j.trsl.2016.08.002 27591027 PMC5718288

[181] Panee J Gerschenson M Chang L: Associations Between Microbiota, Mitochondrial Function, and Cognition in Chronic Marijuana Users. *J Neuroimmune Pharmacol.* 2018;13(1):113–22. 10.1007/s11481-017-9767-0 29101632 PMC5790619

[182] Fulcher JA Hussain SK Cook R: Effects of Substance Use and Sex Practices on the Intestinal Microbiome During HIV-1 Infection. *J Infect Dis.* 2018;218(10):1560–70. 10.1093/infdis/jiy349 29982500 PMC6692862

[183] Hamer HM Jonkers DM Bast A: Butyrate modulates oxidative stress in the colonic mucosa of healthy humans. *Clin Nutr.* 2009;28(1):88–93. 10.1016/j.clnu.2008.11.002 19108937

[184] Holmes E Loo RL Stamler J: Human metabolic phenotype diversity and its association with diet and blood pressure. *Nature.* 2008;453(7193):396–400. 10.1038/nature06882 18425110 PMC6556779

[185] Allen JM Mailing LJ Niemiro GM: Exercise Alters Gut Microbiota Composition and Function in Lean and Obese Humans. *Med Sci Sports Exerc.* 2018;50(4):747–57. 10.1249/MSS.0000000000001495 29166320

[186] Zoetendal EG von Wright A Vilpponen-Salmela T: Mucosa-associated bacteria in the human gastrointestinal tract are uniformly distributed along the colon and differ from the community recovered from feces. *Appl Environ Microbiol.* 2002;68(7):3401–7. 10.1128/AEM.68.7.3401-3407.2002 12089021 PMC126800

[187] Postler TS Ghosh S: Understanding the Holobiont: How Microbial Metabolites Affect Human Health and Shape the Immune System. *Cell Metab.* 2017;26(1):110–30. 10.1016/j.cmet.2017.05.008 28625867 PMC5535818

[188] Louis P Flint HJ: Formation of propionate and butyrate by the human colonic microbiota. *Environ Microbiol.* 2016;19(1):29–41. 10.1111/1462-2920.13589 27928878

[189] Le Poul E Loison C Struyf S: Functional characterization of human receptors for short chain fatty acids and their role in polymorphonuclear cell activation. *J Biol Chem.* 2003;278(28):25481–9. 10.1074/jbc.M301403200 12711604

[190] Frost G Sleeth ML Sahuri-Arisoylu M: The short-chain fatty acid acetate reduces appetite via a central homeostatic mechanism. *Nat Commun.* 2014;5: 3611. 10.1038/ncomms4611 24781306 PMC4015327

[191] De Vadder F Kovatcheva-Datchary P Goncalves D: Microbiota-generated metabolites promote metabolic benefits via gut-brain neural circuits. *Cell.* 2014;156(1–2):84–96. 10.1016/j.cell.2013.12.016 24412651

[192] Smith PM Howitt MR Panikov N: The microbial metabolites, short-chain fatty acids, regulate colonic T _reg_ cell homeostasis. *Science.* 2013;341(6145):569–73. 10.1126/science.1241165 23828891 PMC3807819

[193] Bui TP Ritari J Boeren S: Production of butyrate from lysine and the Amadori product fructoselysine by a human gut commensal. *Nat Commun.* 2015;6: 10062. 10.1038/ncomms10062 26620920 PMC4697335

[194] Macfarlane GT Gibson GR Beatty E: Estimation of short-chain fatty acid production from protein by human intestinal bacteria based on branched-chain fatty acid measurements. *FEMS Microbiol Lett.* 1992;101(2):81–8. 10.1111/j.1574-6968.1992.tb05764.x

[195] Vital M Howe AC Tiedje JM: Revealing the bacterial butyrate synthesis pathways by analyzing (meta)genomic data. *mBio.* 2014;5(2):e00889. 10.1128/mBio.00889-14 24757212 PMC3994512

[196] Thangaraju M Cresci G Itagaki S: Sodium-coupled transport of the short chain fatty acid butyrate by SLC5A8 and its relevance to colon cancer. *J Gastrointest Surg.* 2008;12(10):1773–82. 10.1007/s11605-008-0573-0 18661192

[197] Donohoe DR Collins LB Wali A: The Warburg effect dictates the mechanism of butyrate-mediated histone acetylation and cell proliferation. *Mol Cell.* 2012;48(4):612–26. 10.1016/j.molcel.2012.08.033 23063526 PMC3513569

[198] Trompette A Gollwitzer ES Yadava K: Gut microbiota metabolism of dietary fiber influences allergic airway disease and hematopoiesis. *Nat Med.* 2014;20(2):159–66. 10.1038/nm.3444 24390308

[199] Thorburn AN McKenzie CI Shen S: Evidence that asthma is a developmental origin disease influenced by maternal diet and bacterial metabolites. *Nat Commun.* 2015;6: 7320. 10.1038/ncomms8320 26102221

[200] Lloyd CM Hawrylowicz CM: Regulatory T cells in asthma. *Immunity.* 2009;31(3):438–49. 10.1016/j.immuni.2009.08.007 19766086 PMC3385348

[201] Labbé A Ganopolsky JG Martoni CJ: Bacterial bile metabolising gene abundance in Crohn's, ulcerative colitis and type 2 diabetes metagenomes. *PLoS One.* 2014;9(12):e115175. 10.1371/journal.pone.0115175 25517115 PMC4269443

[202] Ridlon JM Kang DJ Hylemon PB: Bile acids and the gut microbiome. *Curr Opin Gastroenterol.* 2014;30:332–8. 10.1097/MOG.0000000000000057 24625896 PMC4215539

[203] Lefebvre P Cariou B Lien F: Role of bile acids and bile acid receptors in metabolic regulation. *Physiol Rev.* 2009;89(1):147–91. 10.1152/physrev.00010.2008 19126757

[204] Ridlon JM Harris SC Bhowmik S: Consequences of bile salt biotransformations by intestinal bacteria. *Gut Microbes.* 2016;7(1):22–39. 10.1080/19490976.2015.1127483 26939849 PMC4856454

[205] Hartstra AV Bouter KE Bäckhed F: Insights into the role of the microbiome in obesity and type 2 diabetes. *Diabetes Care.* 2015;38(1):159–65. 10.2337/dc14-0769 25538312

[206] Turnbaugh PJ Ley RE Mahowald MA: An obesity-associated gut microbiome with increased capacity for energy harvest. *Nature.* 2006;444(7122):1027–31. 10.1038/nature05414 17183312

[207] Kobayashi M Ikegami H Fujisawa T: Prevention and treatment of obesity, insulin resistance, and diabetes by bile acid-binding resin. *Diabetes.* 2007;56(1):239–47. 10.2337/db06-0353 17192488

[208] Penney NC Kinross J Newton RC: The role of bile acids in reducing the metabolic complications of obesity after bariatric surgery: A systematic review. *Int J Obes (Lond).* 2015;39(11):1565–74. 10.1038/ijo.2015.115 26081915

[209] Patti ME Houten SM Bianco AC: Serum bile acids are higher in humans with prior gastric bypass: potential contribution to improved glucose and lipid metabolism. *Obesity (Silver Spring).* 2009;17(9):1671–7. 10.1038/oby.2009.102 19360006 PMC4683159

[210] Centuori SM Gomes CJ Trujillo J: Deoxycholic acid mediates non-canonical EGFR-MAPK activation through the induction of calcium signaling in colon cancer cells. *Biochim Biophys Acta.* 2016;1861(7):663–70. 10.1016/j.bbalip.2016.04.006 27086143 PMC4900466

[211] Yoshimoto S Loo TM Atarashi K: Obesity-induced gut microbial metabolite promotes liver cancer through senescence secretome. *Nature.* 2013;499(7456):97–101. 10.1038/nature12347 23803760

[212] Bayerdörffer E Mannes GA Ochsenkühn T: Unconjugated secondary bile acids in the serum of patients with colorectal adenomas. *Gut.* 1995;36(2):268–73. 10.1136/gut.36.2.268 7883228 PMC1382415

[213] Coppé JP Desprez PY Krtolica A: The senescence-associated secretory phenotype: the dark side of tumor suppression. *Annu Rev Pathol.* 2010;5:99–118. 10.1146/annurev-pathol-121808-102144 20078217 PMC4166495

